# Surveillance of cardiovascular risk in the normotensive patient with repaired aortic coarctation

**DOI:** 10.1016/j.ijcard.2008.10.043

**Published:** 2010-03-18

**Authors:** Lorna Swan, Mustafa Kraidly, Isabelle Vonder Muhll, Peter Collins, Michael A. Gatzoulis

**Affiliations:** a Adult Congenital Heart Programme, Department of Cardiology, Royal Brompton & Harefield NHS Trust; b National Heart Lung Institute, Royal Brompton Hospital, Imperial College, London, UK

**Keywords:** Coarctation, Blood pressure, Hypertension

## Abstract

**Background:**

Repaired coarctation of the aorta is associated with premature atherosclerosis and an increased risk of cardiovascular events even in normotensive subjects. To date clinical risk stratification has focused on brachial blood pressures ignoring the complex pulsatility of the aortic wave form. The aim of this study was to assess components of this pulsatility in a clinical setting and to suggest possible techniques to improve risk stratification.

**Methods:**

This was a prospective study recruiting patients from a tertiary referral centre. Pulse wave morphology was assessed using applanation tonometry. B-mode ultrasound and cardiac magnetic resonance were used to assess carotid intimal–medial thickness and left ventricular mass.

**Results:**

Forty-six subjects with repaired coarctation of the aorta (range 16–62 years; mean 31 years) and 20 matched controls were studied. Baseline brachial systolic and diastolic blood pressures were not statistically different between the 2 groups. Peripheral (62.5 mmHg (11.3) vs. 50.6 mmHg (15.0), *p* = 0.0008) and central (34.5 mmHg (7.7) vs. 28.7 mmHg (4.7), *p* = 0.005) pulse pressures were elevated in the coarctation patients compared to controls. The reflected pressure wave returned to the ascending aorta earlier in the coarctation group (*p* = 0.007) and the tension time index (TTI) was increased (*p* = 0.03). The sub-endocardial viability index (SVI) was reduced in the coarctation subjects (159 (33) vs. 186 (31)%; *p* = 0.009) but there was no differences in central augmentation index (*p* = 0.35).

**Conclusions:**

This study demonstrates that there are patients with repaired coarctation who have an excellent mid-term outcome free from ventricular hypertrophy, carotid intima medial thickening and with relatively preserved vascular reactivity. However even in this “best outcome” cohort there were abnormal vascular characteristics that may predispose to increased cardiovascular risk. Simple non-invasive investigations can more extensively characterise these sub-clinical abnormalities and by utilised in long-term surveillance.

## 1 Introduction

In recent years our understanding of coarctation of the aorta has changed significantly. What was previously perceived to be a simple localised abnormality of the descending aorta is now acknowledged to be a more complex lesion with life-long sequelae [1–3]. Widespread vascular abnormalities, propensity towards end-organ damage and accelerated atherosclerosis all contribute to the long-term morbidity and mortality associated with this condition. Indeed the most common cause of death in a post-repair cohort is myocardial infarction which can occur at a relatively young age [4–6].

Despite this knowledge little has changed regarding the care of this patient group in adulthood. Late management remains “reactive” and there is only a patchy understanding of effective primary prevention. The foundation of long-term follow-up remains the assessment of baseline blood pressure in an outpatient or practice setting. Such measurements may however be a poor surrogate for the complex pulsatile nature of the aortic blood pressure which results from the interplay of vessel wall characteristics, flow dynamics and ventricular function [7]. In addition end-organ damage can also occur in post-repair patients despite normal clinic blood pressures [8].

The aim of this study was to assess a group of well and asymptomatic patients with repaired coarctation. The study endeavoured to apply simple non-invasive tests to assess if more detailed risk stratification could be performed in a manner applicable to routine clinical practise.

## 2 Materials and methods

### 2.1 Subjects

Adults with repaired coarctation were recruited prospectively from the Adult Congenital Heart Clinic at the Royal Brompton Hospital, London. Exclusion criteria included pregnancy, known systemic hypertension, other hemodynamically significant cardiac lesions, previous complex congenital heart disease, recoarctation awaiting intervention and the current use of vasoactive medication. A corresponding matched cohort of control subjects was recruited from hospital staff. The study protocol was approved by the institution's ethics committee.

Patients were studied in a fasting state. Bloods were taken for routine biochemistry including serum lipids and blood glucose. A brief self-reported questionnaire assessed smoking habits and family history. Clinical examination was performed including three measurements of resting supine blood pressure in each limb using an automated calibrated blood pressure monitor (Dinamap, Criticon, Tampa, Florida).

### 2.2 Tonometry

**Fig. 1:**
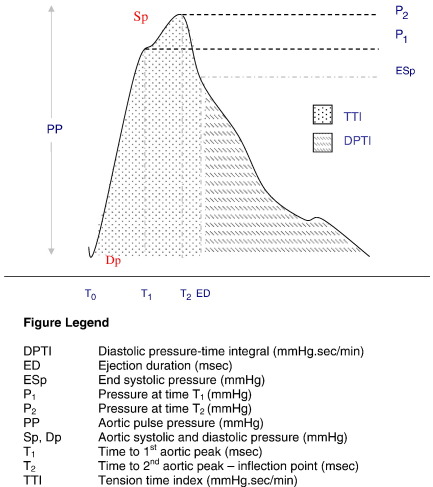
Schematic of aortic arterial pulse.

Peripheral pressure waveforms were recorded from the right radial artery using applanation tonometry (SphygmoCor PX Version 6.31, ATCOR, Sydney, Australia) [9]. Brachial blood pressure (BP) was measured non-invasively every 5 min throughout the study period (Dinamap). Immediately after each BP measurement, radial artery tonometry recording were taken and data collected directly into a laptop (Toshiba Satellite 2140CDS). After 20 sequential waveforms had been acquired, an averaged peripheral waveform was produced. A corresponding central aortic waveform, derived using a validated transfer function [10], was then generated to determine augmentation index (AIa) and central pressure. Augmentation index (AIa), was calculated as the augmented pulse pressure (P2–P1) divided by the pulse pressure (PP) × 100 [13] (see Fig. 1). Ejection duration (the time from the foot of the pressure wave to the incisura) and the timing of the reflected wave (T2 defined from the foot of the pressure wave to the inflection point) [11,12] were also assessed.

The area under the systolic portion of the pressure waveform (tension time index — TTI) and the diastolic pressure–time integral (DPTI) were also determined. From these variables DPTI divided by TTI was calculated. This is a ratio between the diastolic portion of the aortic blood pressure waveform and the systolic component. This description of the perfusion pressure of the coronary arterial bed during diastole versus the systolic pressure load seen by the left ventricle is thought to be a marker of work-supply balance (sometimes referred to as the subendocardial viability index) [14].

Endothelial-dependent and independent vascular function was assessed using inhaled salbutamol (400 mcg) and sublingual glyceryl trinitrate (250 mcg) challenges [12].

### 2.3 B-mode carotid ultrasound

Carotid scans were performed using an Acuson Sequioa ultrasound machine with an 8 MHz transducer. Following the acquisition of the scan the diastolic images which best demonstrated intimal medial thickening (IMT) were analysed. Both near and far walls of the common carotid artery were visualised on the same scan to ensure the transducer was transecting the artery at 90°. Scans were read by a single observer blinded to other results. Images were analysed using a quantitative analysis package (Siemens) giving an axial resolution of 0.001 mm. Measurements of IMT were made at 1 mm intervals over a 10 mm segment of vessel. The maximal and mean IMT measurements were determined for the near and far walls of the left and right common carotid arteries [15].

### 2.4 Magnetic resonance imaging

Left ventricular mass was calculated from a short axis cine stack. Gated cardiac magnetic resonance images were acquired using a Siemens 1.5 T scanner and analysed by an experienced imager who was blinded to the other study results. Volume and mass data was analysed from the short axis stack by manually delineating the endocardial boundary in systole and end-diastole [16].

### 2.5 Statistics

Descriptive data for the cohort are presented as mean values and standard deviations. Non-parametric variables are described using medians. Differences between groups were compared using an unpaired *t*-test except for non-parametric variables, which were analysed using a Mann–Whitney test (GraphPad InStat, GraphPad Software, Inc). Variables were compared using Pearson's correlation coefficients (*r*). A *p* value of less than 0.05 was taken to be statistically significant.

## 3 Results

Forty-six coarctation subjects (24 males: 22 females) and 20 controls (11 males: 9 females) were recruited over 14 months. The mean age of the patients was 31 years (range 16–62 years). Age at the time of first coarctation repair was 10.7 (10.1) years (range first day of life to 35 years). The intervention techniques employed were end-to-end anastomosis (*n* = 19); patch repair (*n* = 8); subclavian flap (*n* = 3); tube graft (*n* = 3); primary angioplasty (*n* = 4) and other/unknown (*n* = 9). Twenty-six percent of the cohort were repaired in the first 2 years of life and eleven subjects had undergone more than one coarctation repair procedure.

### 3.1 Baseline risk factors

None of the coarctation subjects had a history of hypertension, diabetes or hyperlipidaemia. There was no personal history of ischaemic heart disease or stroke. Baseline systolic and diastolic blood pressures were normal and there was no excess of traditional cardiovascular risk factors (Table 1).

Twenty five (54%) of the coarctation group had a bicuspid aortic valve. No subject had hemodynamically significant valve disease. There was no evidence of significant recoarctation in any subject.

### 3.2 Blood pressure pulsatility and central hemodynamic

The tonometry-derived measures of pulsatility and central aortic pressures are presented in Table 2. Central pulse pressure was related to current age (*r* = 0.41, *p* = 0.02) but the time to the aortic reflective wave (T2) was not.

There was no difference between the coarctation subjects and the control population in terms of simple measurements of vascular reactivity (Table 3). This was true for both measurement of global endothelial function (% change in central augmentation index following 400 mcg salbutamol) and the assessment of nitric oxide-independent smooth muscle reactivity (% change following 250 mcg GTN).

### 3.3 Carotid intima media thickness

In this cohort there was no significant difference in any of the intima media thickness parameters, a surrogate marker for coronary atheroma [17], between the coarctation subjects and the controls — mean far wall IMT right common carotid 0.55 vs. 0.5 mm, *p* = 0.26 (coarctation vs. control); mean far wall IMT left common carotid 0.59 vs. 0.54 mm, *p* = 0.27). The major determinants of carotid IMT were central systolic blood pressure (*r* = 0.74, *p* < 0.001) and central pulse pressure (*r* = 0.42, *p* = 0.02).

### 3.4 Left ventricular mass

There was a trend towards an increased left ventricular mass in this selected population of repaired coarctation subjects (76.5 (14.5) vs. 71.6 (9.7) g/m sq., *p* = 0.21) but this was not statistically significant. There was no relationship between LV mass and IMT (*r* = − 0.05, *p* = 0.79) in this cohort.

## 4 Discussion

Coarctation of the aorta is associated with late cardiovascular events with myocardial infarction and cerebrovascular disease accounting for the majority of deaths. This association is present even in individuals who had a successful repair. For the cardiologist caring for an adult with repaired coarctation a clinical difficulty is the early identification of those at increased risk of subsequent cardiovascular events and in the determination of effective primary prevention.

Recently it has become clear that simple brachial systolic–diastolic blood pressure parameters may be inadequate to describe the variability of the aortic pressure wave — exemplified by the fact that in early systole cardiac output can be 6 times greater than mean flow with only a relatively narrow pulse pressure range [7]. Changes in this system, for example age-related aortic wall stiffening, can detrimentally affect LV systolic loading [18]. Pulse pressure is known to be an independent predictor of cardiovascular outcome. In the SAVE (Survival and Ventricular Enlargement study) and the SOLVD (Studies of Left Ventricular Dysfunction) cohorts an increase in pulse pressure of 10 mmHg was associated with a 5% increase in mortality [19,20].

The cohort investigated in this study represents the “best” end of the coarctation spectrum — repaired patients with no evidence of recoarctation or systemic hypertension. Our data reassuringly showed that even out to the age of 30 years (20 years post-repair) that increased intimal medial thickening and left ventricular hypertrophy are not inevitable in this population. In contrast to other less select cohorts significant endothelial dysfunction and smooth muscle vascular impairment were not ubiquitous [21,22].

In addition there is no excess of traditional cardiovascular risk factors and no change in Framingham risk scores.

Despite these positive features this cohort still demonstrated abnormal central pulse pressures. In addition the reflected wave returned earlier either due to an increase in pulse wave velocity or a more proximal reflection point. This mirrors the finding from invasive assessment described by Murakami et al. [23]. An increased pulse pressure and an early reflective wave can lead to an elevated end-diastolic pressure [24,25]. Many of these detrimental hemodynamics can by summarised in the subendocardial viability index that demonstrates an imbalance between loading and perfusion — a potential precursor of myocardial ischaemia [26,27]. Asymptomatic myocardial perfusion detects have been reported in repaired coarctation patients utilising nuclear perfusion techniques [28].

In subjects with repaired coarctation vascular compliance has both structural and functional components [29]. The structural component is difficult to ameliorate but pharmacological intervention may be able to modify functional abnormalities. Whether these will have a long-term preventative role, inhibiting the development of left ventricular hypertrophy and atheroma should be the subject of future investigation.

Previous studies have demonstrated abnormalities in pulse wave velocity and flow-mediated dilatation following coarctation repair [21,22]. The advantage of applanation tonometry is that it is a simple, portable investigation suited to the clinical environment. Serial tonometry could be performed during routine follow-up or before and after intervention (pharmaceutical or catheter-based stenting).

### 4.1 Limitations

This study has the limitations of a cross-sectional design. Patients were clinically defined as being normotensive if not previously diagnosed and on no therapy. Patients may however have had mildly elevated blood pressure profiles on 24-hour testing. In addition subtle effects of different repair technique, minor residual repair site gradients or the presence of a bicuspid aortic valve on proximal hemodynamics cannot be excluded.

The general transfer factor used to assess central hemodynamics has not been specifically validated in patients with coarctation repair and therefore small differenced in central pressures may be possible. In addition tonometry may under-estimate augmentation index and be more susceptible to error [30]. Further longitudinal studies, including coarctation patients with hypertension, will be required to further investigate the pathogenesis of abnormal aortic pulsatility following repair.

## 5 Conclusions

This study demonstrates that there are patients with repaired coarctation who have an excellent mid-term outcome free from ventricular hypertrophy, carotid intima medial thickening and with relatively preserved vascular reactivity. However even in this “best outcome” cohort there were abnormal vascular characteristics that may predispose to increased cardiovascular risk. Simple non-invasive investigations can more extensively characterise these sub-clinical abnormalities and thus more effectively target and monitor future preventative therapies.

## Figures and Tables

**Table 1:** Coarctation subjects vs. controls — baseline data (mean (SD)).

	Coarctation	Control	*p*
Numbers	46	20	
Male:female	24M:22F	11M;9F	
Age (years)	31.0 (9.5)	33.2 (6.8)	0.36
Family history of hypertension	14/46 (30.4%)	7/20 (35%)	0.36
Family history of ischaemic disease	19/46 (41%)	7/20 (35%)	0.78
Smoker (current)	3/46 (6.5%)	1/20 (5%)	0.58
Weight (kg)	73.7 (19.3)	75.5 (14.1)	0.7
Height (cm)	168.9 (19.7)	174.6 (12.3)	0.24
Body mass index (kg/m^2^)	24.3 (4.6)	24.2 (2.9)	0.92
Brachial systolic BP (mmHg)	121 (11.8)	115.5 (12.2)	0.13
Brachial diastolic BP (mmHg)	69.2 (7.9)	71.4 (8.1)	0.34
Brachial mean BP (mmHg)	85.4 (9.0)	85.0 (8.0)	0.87
Arm-leg gradient (mmHg)	− 4.1 (16.9)	− 19.9 (15.7)	0.002
Total cholesterol (mmol/l)	4.8 (1.0)	4.6 (0.63)	0.57
HDL (mmol/l)	1.48 (.41)	1.37 (0.41)	0.3
Glucose (mmol/l)	5 (0.48)	5.24 (0.87)	0.17

**Table 2:** Coarctation subjects vs. controls — tonometry data (mean (SD)).

	Coarctation	Control	*p* value
Number	46	20	
Heart rate (bpm)	63.8 (10.3)	60.3 (9.0)	0.22
Peripheral pulse pressure (mmHg)	62.5 (11.3)	50.6 (15)	0.0008
Central systolic BP (mmHg)	104.6 (10.1)	101.0 (10.6)	0.24
Central diastolic BP (mmHg)	70.1 (7.9)	72.2 (8.3)	0.36
Central mean BP (mmHg)	84.8 (8.0)	85.4 (8.0)	0.81
Central pulse pressure (mmHg)	34.5 (7.7)	28.7 (4.7)	0.005
Central 1st pressure peak (mmHg)	99.5 (8.8)	98.5 (10.1)	0.56
Central 2nd pressure peak (mmHg)	103.6 (10.6)	101 (10.5)	0.56
Ejection duration (ms)	331 (22.0)	325 (24.3)	0.41
Timing of reflected wave (ms)	149.9 (15.4)	162.6 (17.4)	0.007
Central augmentation index (%)	115.4 (18.8)	111.0 (13.2)	0.35
Tension time index	2050 (335)	1847 (307)	0.03
Diastolic pressure–time integral	3157 (500)	3282 (356)	0.33
Central sub-endocardial viability index (%) DTPI/TTI	159.6 (33.3)	185.7 (31.3)	0.009

**Table 3:** Augmentation index (AI) response to B-agonist and nitrate challenge.

	Coarctation	Control	*p* value
Number	46	20	
Central AI — baseline	115.6 (18.6)	111.0 (13.1)	0.34
Central AI — post salbutamol	112.1 (17.5)	104.9 (10.1)	0.10
Central AI — post GTN	94.8 (10.6)	92.9 (6.6)	0.49
% change in central AI — salbutamol	− 3.77 (5.6)	− 5.0 (7.2)	0.47
% change in central AI — GTN	− 20.1 (16.5)	− 15.5 (8.8)	0.26
